# Non-inferiority of cleavage-stage versus blastocyst-stage embryo transfer in poor prognosis IVF patients (PRECiSE trial): study protocol for a randomized controlled trial

**DOI:** 10.1186/s12978-020-0870-y

**Published:** 2020-01-30

**Authors:** Werner M. Neuhausser, Denis A. Vaughan, Denny Sakkas, Michele R. Hacker, Tom Toth, Alan Penzias

**Affiliations:** 1 Department of Obstetrics and Gynecology, Beth Israel Deaconess Medical Center, Boston, MA USA; 2 Department of Obstetrics, Gynecology and Reproductive Biology, Harvard Medical School, Boston, MA USA; 3 Boston IVF, Waltham, MA USA; 4 Department of Stem Cell and Regenerative Biology, Harvard University, Cambridge, USA; 5 Broad Institute of MIT and Harvard, Cambridge, USA

**Keywords:** In vitro fertilization, Cleavage-stage transfer, Blastocyst transfer, Non-inferiority randomized controlled trial

## Abstract

**Background:**

With improvements in in vitro culture techniques there has been a steady shift in practice to transfer embryos at the blastocyst stage (post fertilization day (p.f.d.) 5–7), when embryos reach the endometrial cavity during natural conception. For patients with > 5 zygotes on day 1 of embryo development, fresh blastocyst embryo transfer (ET) increases live birth rates when compared to cleavage stage (p.f.d. 3) transfer. In poorer prognosis patients (≤ 5 zygotes) cleavage stage ET is commonly performed to reduce the risk of cycle cancellation if no embryo survives to the blastocyst stage. However, there is a dearth of randomized controlled trial (RCT) data demonstrating improved live birth rates per cycle for cleavage vs blastocyst stage ET in this subgroup of patients. The hypothesis of the PRECiSE (PooR Embryo Yield Cleavage Stage Versus blaStocyst Embryo Transfer) trial is that blastocyst ET is not inferior to cleavage stage ET with regard to live birth rates per retrieval in poorer prognosis patients. The adoption of routine blastocyst culture for all patients would result in higher rates of single embryo transfers (SET), reduced incidence of multiple pregnancies and simplified laboratory protocols, thereby reducing costs.

**Methods/design:**

Multicenter, non-inferiority randomized controlled trial (RCT) comparing blastocyst to cleavage stage embryo transfer in poorer prognosis patients with ≤5 zygotes on day 1 after fertilization. The primary outcome is live birth per retrieval. Secondary outcomes include: time to pregnancy, clinical pregnancy, ongoing pregnancy, miscarriage and multiple pregnancy rate (per retrieval). This trial will enroll 658 women with ≤5 zygotes on day 1 at 6 IVF centers over the course of 22 months.

**Discussion:**

If the hypothesis is proven true, the data from this trial may facilitate the adoption of uniform blastocyst culture in all IVF patients.

**Trial registration:**

ClinicalTrials.gov Identifier: NCT03764865. Registered 5 December 2019, Protocol issue date: 4 December 2018, Original.

## Plain English summary

Embryos created with assisted reproductive technology (ART, e.g. in vitro fertilization (IVF)) can be transferred into a woman’s uterus at either the cleavage (p.f.d. 3) or the blastocyst stage (p.f.d. 5–7). It is thought that embryos that develop into blastocysts in culture are more likely to be viable inside the uterus and result in a successful pregnancy than a cleavage stage embryo. In vitro culture beyond p.f.d. 3 therefore allows for self-selection of embryos that successfully reach the blastocyst stage. This allows the transfer of fewer embryos and decreases the likelihood of multiples (twins, triplets, etc.). However, it is possible that culture of embryos to the blastocyst stage in the lab leads to the loss of some embryos that may have survived inside the uterus. Thus, at many centers, cleavage-stage transfer is performed in patients with few available embryos to reduce the incidence of cycle cancellation if no embryo reaches the blastocyst stage. On the other hand, transferring a blastocyst on p.f.d. 5–7 improves uterine/embryonic synchronicity and may thereby improve outcomes. For poorer prognosis patients with few embryos, no high-quality studies have evaluated whether blastocyst transfer increases live birth rates per retrieval compared to cleavage-stage transfer. As a result, a clinical dilemma exists for the timing of embryo transfers in these patients. The purpose of this study is to assess IVF outcomes among patients with ≤5 embryos on day 1 after fertilization (zygotes) who have a cleavage-stage transfer compared with those who have a blastocyst transfer.

## Background

Since the first in vitro fertilization (IVF) pregnancy in 1978, IVF has evolved into an effective treatment for many subfertile couples or women in whom less invasive treatment methods have failed or are unlikely to be effective. Embryos created through IVF generally are transferred into the uterus at either the cleavage stage (p.f.d. 3 after oocyte retrieval) or blastocyst stage (p.f.d. 5–7 after oocyte retrieval). Early in the history of IVF, most providers transferred p.f.d 2 or 3 embryos, because the available culture media and techniques led to low blastocyst stage development in vitro. However, with improvements in in vitro culture methods, there has been a steady shift in practice to transfer embryos at the blastocyst stage, which mirrors more closely when embryos reach the endometrial cavity during natural conception.

A recent Cochrane meta-analysis of 27 randomized controlled trials (RCTs) by Glujovsky et al. found a higher live birth rate, per transfer, in the fresh blastocyst transfer group compared to cleavage-stage transfer (odds ratio (OR) 1.48, 95% confidence interval (CI) 1.20 to 1.82) and no evidence for a difference in the rates of miscarriage, multiple pregnancies, and high-order multiples [1]. However, this analysis only included 539 patients and was not powered to identify subgroups of patients who may benefit from a cleavage-stage transfer. In addition, because of attrition during culture to the blastocyst stage in vitro, the live birth rate should be evaluated per retrieval, rather than per transfer. Based on individual RCTs, the best evidence for an increased likelihood of live birth after transfer of fresh blastocysts compared with cleavage-stage embryos exists in good prognosis patients (defined by such factors as age, number of previous failed attempts, ovarian response, and number and quality of embryos) [1–6]. As a result, there now is a general consensus that, for good prognosis patients, it is beneficial to transfer a blastocyst rather than a cleavage stage embryo. However, in unselected patients, RCTs have yielded conflicting results, and in poorer prognosis patients no high-quality studies have evaluated whether the live birth rate is higher with fresh blastocyst or cleavage-stage transfer [1, 7]. Thus, at present, many providers offer a p.f.d 3 transfer to patients with few available embryos to reduce the incidence of cycle cancellation due to failure of embryo development to the blastocyst stage. At our center, this approach is offered to patients with ≤5 zygotes on day 1 after fertilization and as a result 35% of all patients receive a p.f.d 3 embryo transfer, which is consistent with national trends.

Although there is evidence that blastocyst transfer in fresh cycles yields higher live birth rates in good prognosis patients, it remains unclear whether the day of transfer affects the live birth rate. Glujovsky et al. did not find a difference in the live birth rate per retrieval between cleavage-stage and blastocyst transfer (OR 0.89, 95% CI 0.64 to 1.22) in their meta-analysis [1]. However, there was significant heterogeneity among the RCTs, with some showing benefit with blastocyst and others with cleavage-stage transfer. In addition, almost all of these studies used the conventional slow freeze technique for embryo cryopreservation, which provides a lower survival rate of embryos compared with the new technique of vitrification. The only RCT that used vitrification reported a benefit of blastocyst transfer for the live birth rate per retrieval (OR 2.44, 95% CI 1.17 to 5.12) when at least 4 zygotes were obtained [8]. Thus, new studies reporting live birth rates per retrieval, as well as other adverse outcomes, are urgently needed, particularly in poorer prognosis patients.

### Risks and benefits of cleavage stage vs blastocyst stage embryo transfer

Blastocyst transfer offers several theoretical advantages over traditional cleavage-stage transfer. Culture of embryos to p.f.d. 5 allows for self-selection of embryos, meaning those that develop into a blastocyst in vitro are more likely to be viable in vivo and result in a viable pregnancy. Thus, blastocysts have higher implantation potential compared with cleavage stage embryos and provide the opportunity to select the most viable embryo(s) for transfer, thus decreasing the number of embryos being transferred and, as a consequence, the likelihood of multiples (twins, triplets, etc.). In addition, because human embryos reach the uterine cavity on day 4–5 of development, the endometrium may not provide the appropriate physiological environment for cleavage stage embryos, particularly in the setting of ovarian stimulation and elevated estrogen. Thus, transferring at the blastocyst stage improves uterine/embryonic synchronicity and may improve outcomes. In addition, the logistics of orchestrating transfers at different developmental stages imposes a considerable workload on embryology labs, thereby increasing costs that could be avoided if all embryo transfers occurred at the blastocyst stage. However, it is possible that the attrition of p.f.d. 3 embryos in vivo is lower than the attrition in vitro and that blastocyst transfer leads to the loss of embryos that may have survived in vivo*,* resulting in reduced pregnancy rates per retrieval.

In addition, culture to p.f.d. 5–7 is expected to result in higher incidence of cycle cancellation and lower rates of embryo cryopreservation due to failure of embryos to develop into a blastocyst [9] [10]. Cycle cancellation rates have been shown to be higher for blastocyst transfers than cleavage-stage transfers (OR 2.50, 95% CI 1.76 to 3.55) [1]. Whilst transfer of cleavage stage embryos might benefit poorer prognosis patients, it also may be associated with a range of adverse consequences. Cleavage-stage transfer may result in an increased incidence of biochemical pregnancies, miscarriages and multiples as more embryos generally are transferred on p.f.d. 3 compared to the blastocyst stage. Therefore, a clinical dilemma exists regarding when to transfer embryos in poorer prognosis patients. Currently, many practices in the United States transfer embryos from these patients on p.f.d. 3, in large part due to fear of cycle cancellation and thus eliminating any possibility of pregnancy. Retrospective data from Boston IVF, BIDMC’s affiliated infertility treatment center, demonstrate that the live birth rate per transfer among poorer prognosis patients is 24% among those who have a cleavage-stage transfer and 38% among those who have a blastocyst transfer. The transfer cancellation rate of ~ 5% in those slated for blastocyst transfer is significantly higher than for p.f.d. 3 transfer (unpublished data). However, the ultimate goal of assisted reproductive technology (ART) is to achieve a live birth, not an embryo transfer, and the choice of cleavage-stage or blastocyst embryo transfer for poorer prognosis patients generally is based on clinical intuition and experience as well as patient preference. Further, patients with unsuccessful p.f.d. 3 transfers will have used supplemental progesterone for 2 weeks and may wonder if the uterine lining was at fault rather than the embryo. This may lead to an unnecessary investigation of endometrial issues. Other practices choose to grow embryos to p.f.d. 5–7 and if none are viable for transfer assume that pregnancy would not have occurred with a p.f.d. 3 transfer. Conversely, patients who do not have an embryo transfer may wonder if their embryo might have done better in their uterus than during culture in vitro.

Our hypothesis for this study is that there is no clinically significant difference in live birth rates between cleavage stage and blastocyst stage transfer in poorer prognosis patients. This hypothesis is based on (1) the established observation that most embryos failing to progress to the blastocyst stage will be chromosomally abnormal [11, 12] and will not result in a live birth even if transferred on p.f.d. 3 (2) the idea that beneficial interactions between cleavage stage embryos and the reproductive system are likely to occur in the Fallopian tube and will not occur after uterine transfer. Thus, the primary aim of this study is to test the hypothesis that the live birth rate among poorer prognosis patients who are randomized to blastocyst stage embryo transfer is not inferior to the live birth rate of patients who are randomized to cleavage-stage embryo transfer.

## Methods/design

### Aim

**Fig. 1:**
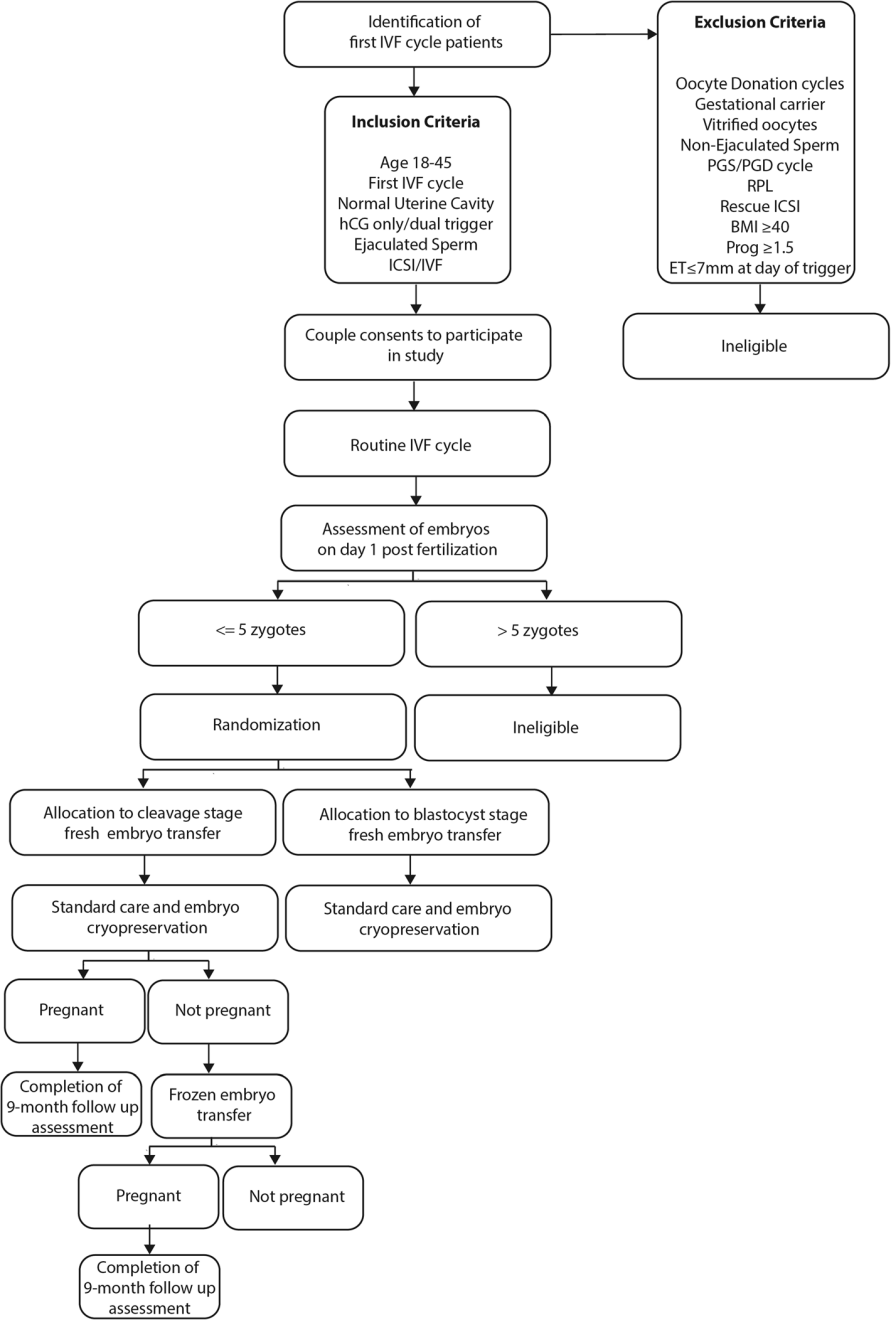
Study design flow diagram. First cycle IVF patients meeting inclusion criteria will be consented to participate in the study and undergo an autologous IVF cycle. Patients who consent to participate in the study may be determined ineligible prior to randomization depending on the number of available embryos on day 1 post fertilization. After allocation to cleavage-stage or blastocyst-stage fresh embryo transfer patients will receive standard care and any embryos not transferred will be cryopreserved at the blastocyst stage. If pregnancy is not achieved in the fresh transfer cycle any remaining frozen embryos will be transferred. All pregnant patients will be followed and pregnancy outcomes recorded. To investigate pregnancy outcomes per IVF retrieval we will follow participants until all cryopreserved embryos have been transferred or a transfer results in a live birth, whichever occurs first

The primary aim of this trial is to test the hypothesis that blastocyst embryo transfer in poorer prognosis IVF patients is non-inferior to cleavage-stage transfer with regard to the live birth rate per retrieval. This international, prospective, two-arm non-inferiority RCT will compare pregnancy outcomes per IVF retrieval among 658 patients with ≤5 zygotes on day 1 of embryo development randomized to either cleavage- or blastocyst-stage fresh embryo transfer. We will conduct this RCT at Boston IVF, a large infertility treatment center covering several states in the US and Clinica Eugin, one of the largest IVF providers in Europe based in Barcelona, Spain. Figure 1 depicts the flow of study participants.

### Study design and setting

Eligible patients will be counseled extensively by the physician of record, as is usual clinical practice, on the merits of p.f.d. 3 versus p.f.d. 5 embryo transfer and will be made aware of the study and given information sheets describing the study. If the couple is interested in participating, a member of the study team will discuss the study in detail and obtain written, informed consent prior to the start of the IVF cycle or early on in the cycle. In particular, the study team will inform the patient that for ≤5 zygotes available on p.f.d. 1, we estimate that the risk of not having an embryo to transfer is approximately 5% and increases as the number of embryos diminishes. Patients will also be counseled that embryos arresting in vitro may have a higher or lower likelihood of resulting in a live birth if they were transferred on p.f.d. 3 versus p.f.d. 5.

**Fig. 2:**
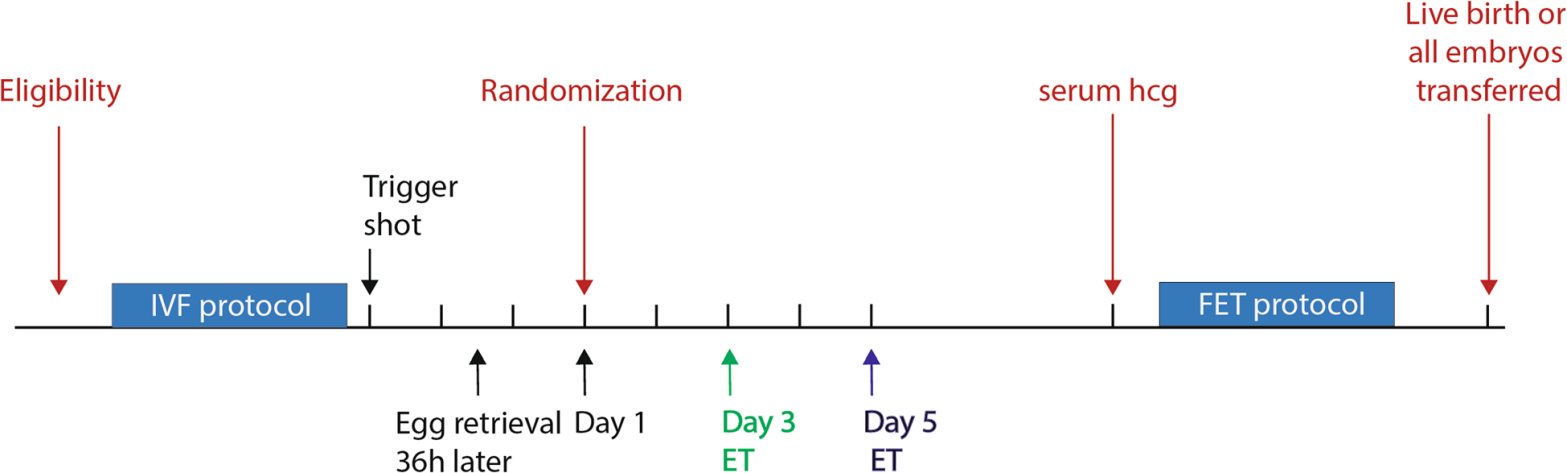
Study protocol. IVF stimulation protocols, trigger agents (hCG) and fertilization (IVF or intracytoplasmic sperm injection) will follow standard clinical practice. Patients will be randomized to p.f.d. 3 or p.f.d. 5 ET on day 1 of embryo development if they have ≤5 embryos available. Selection of embryos will be based on morphology and the number of embryos transferred will be based on the current American Society of Reproductive Medicine (ASRM) guidelines. Following the embryo transfer, clinical care will follow standard practice. Participants will receive vaginal progesterone for luteal support starting the day after egg retrieval until 8 weeks gestation or until 10 days after fresh embryo transfer, if the serum pregnancy test is negative. Any unused embryos will be cultured to p.f.d. 5–7 and cryopreserved by vitrification per standard clinical protocols. A pregnancy test (serum hCG) will be performed on day 10 following embryo transfer. If negative, any cryopreserved embryos will be transferred in subsequent frozen embryo transfer cycles as per standard protocol. We will administer intra-muscular progesterone with or without vaginal progesterone for luteal support in thaw cycles after the endometrial lining reaches a minimal trilaminar endometrial thickness of 7 mm (=day 1) with embryo transfer in the afternoon of day 6

Study participation will not influence any element of treatment other than the day of embryo transfer. Stimulation protocols, trigger agents (hCG) and fertilization (IVF or intracytoplasmic sperm injection) will follow standard clinical practice. Participants may take supplements such as Co-enzyme Q10 and DHEA during stimulation as recommended by their physician. Participants will receive vaginal progesterone (Crinone 90 mg daily) for luteal support starting the day after egg retrieval until 8 weeks gestation or until 10 days after fresh embryo transfer, if the serum pregnancy test is negative. Selection of embryos will be based on morphology according to current embryology laboratory selection protocols. The number of embryos transferred will be based on the current American Society of Reproductive Medicine guidelines [13]. Patients will be randomized to p.f.d. 3 or p.f.d. 5 ET on day 1 of embryo development if they have ≤5 embryos available. Following the embryo transfer, clinical care will follow standard practice. Any unused embryos will be cultured to p.f.d. 5–7 and cryopreserved by vitrification per standard clinical protocols. It is standard practice at our center to cryopreserve only good-quality blastocysts (inner cell mass and trophectoderm grades of BB or better according to the Gardner grading system on p.f.d. 5 or 6 (rarely p.f.d. 7) [14]. A pregnancy test (serum hCG) will be performed on day 10 following embryo transfer. If negative, any cryopreserved embryos will be transferred in subsequent frozen embryo transfer cycles as per standard protocol. To investigate pregnancy outcomes per IVF retrieval we will follow participants until all cryopreserved embryos have been transferred or a transfer results in a live birth, whichever occurs first. In accordance with standard clinical practice at our center, we will use exogenous estrogen and progesterone for endometrial preparation prior to cryopreserved blastocyst transfer for both treatment arms. Each patient will receive a course of combined oral contraceptive pills followed by oral estradiol (6 mg daily), which is started on day 2 of the withdrawal bleed. We will administer intra-muscular progesterone with or without vaginal progesterone for luteal support in thaw cycles after the endometrial lining reaches a minimal trilaminar endometrial thickness of 7 mm (day 1) with embryo transfer in the afternoon of day 6 [15]. No drugs or new devices will be examined as part of this study. The study protocol follows the SPIRIT reporting guidelines [16] and is depicted in Fig. 2.

### Outcome measures

The primary study endpoint is live birth per retrieval, defined as delivery of a live born infant ≥22 weeks of gestation. The secondary outcomes will include: clinical pregnancy rate per retrieval, defined by confirmation of a gestational sac on ultrasound; ongoing pregnancy rate per retrieval, defined by ultrasound confirmation of a gestational sac with at least one fetal pole with a fetal heartbeat; miscarriage rate per retrieval; multiple pregnancy rate per retrieval and time from randomization to pregnancy per retrieval. In addition, we will record the outcomes for any embryos created but not transferred in this stimulation cycle, to calculate the live birth rate per retrieval. All data that are collected for the trial are clinical data that will be stored in the electronic medical record as part of routine clinical care.

### Study population

All women age 18 to 44 years who present for their *first* autologous IVF cycle will be potentially eligible to participate. Additional eligibility criteria include providing written, informed consent.

Women will not be eligible to participate base on the exclusion criteria below

- Planned preimplantation genetic testing
- History of recurrent pregnancy loss (≥2 spontaneous abortions)
- Treatment plan indicates preference for either p.f.d. 3 or p.f.d. 5 embryo transfer
- Planned gestational carrier
- Body mass index > 40
- Presence of uterine factor infertility

In addition, participants will be withdrawn from the study after consent and before randomization if they meet any of the criteria below during the course of their IVF cycle:

- > 5 or < 1 embryos with 2 pronuclei on day 1 after egg retrieval
- Endometrial lining < 7 mm measured on the day of trigger
- Lupron-only trigger
- Elevated progesterone in the fresh cycle (≥1.5 ng/ml)
- Delayed fertilization (> 18 h)
- Rescue intracytoplasmic sperm injection (following failed regular fertilization)
- The use of non-ejaculated sperm (testicular sperm extraction)
- Embryo transfer number outside American Society of Reproductive Medicine (ASRM) guidelines
- Cycle is converted to a cycle in which all embryos are frozen

### Randomization

Embryos will be assessed on day 1 following egg retrieval, in accordance with usual clinical practice. If patients have 1 to ≤5 embryos with 2 pronuclei available, they will be randomized to receive a cleavage- or blastocyst -stage embryo transfer. We will use computer-generated block randomization to randomize participants in a 1:1 ratio to the day 3 or day 5 treatment arm. Randomization will be stratified based on treatment center, age (< 38 and ≥ 38 years) and by embryo number (1–2 and 3–5 embryos on day 1 after egg retrieval). Randomization will be performed electronically using a secure web application (REDCap). Allocation concealment will be ensured, as randomization will not occur until the patient becomes eligible to participate in the trial, which takes place after consent to participate. In addition, randomization will be performed by embryology lab personnel and not recruiting study physicians. As per standard clinical practice, a clinician (who also is a member of the study team) will call participants to inform them of the number of fertilized embryos and, at this point, will also inform them of the day of transfer to which they have been randomized. At this point, participants will have an opportunity to not continue with the day of transfer to which they were randomized. We anticipate that most of the crossovers will occur due to participants in the p.f.d. 5 group with only 1–2 embryos wishing to have an embryo transfer on p.f.d. 3. Participants who choose to not continue with their assigned day of transfer will be asked to continue their participation so that we can collect cycle outcome data for the intention-to-treat analysis. Due to the nature of the intervention neither participants nor staff can be blinded to allocation, but are strongly inculcated not to disclose the allocation status of the participant at the follow up assessments. An employee at Boston IVF outside the research team will feed data into the computer in separate datasheets so that the researchers can analyze data without having access to information about the allocation. Trial conduct and protocol adherence will be audited every 6 months by the clinical director of each individual study site.

### Rationale for the non-inferiority hypothesis and for sample size estimation

Given the potential benefits of blastocyst-only transfers and the fact that cleavage-stage embryo transfer for patients with few viable embryos is currently routine practice at many IVF centers, empirical evidence is needed to determine the value of blastocyst transfer compared to cleavage-stage transfer in poorer prognosis patients. Most human embryos not resulting in a pregnancy fail due to inherent chromosomal abnormalities that cannot be rescued by the environment the embryo encounters. In addition, plausible arguments exist for better developmental outcomes for both p.f.d. 3 transfer and p.f.d. 5 transfer. Earlier exposure of embryos to an in vivo environment may maximize developmental outcomes of cleavage-stage embryos. On the other hand, the improved temporal-spatial synchrony between the embryo and uterine cavity supports blastocyst transfers. Thus, this question lends itself to a non-inferiority design.

Our choice of a non-inferiority margin, i.e. the largest clinical difference in the live birth rate per retrieval that is acceptable between cleavage- and blastocyst-stage embryo transfer, is based on a combination of clinical judgment and statistical reasoning. Given that there are no data from previous trials to help define the clinical difference between cleavage-stage and blastocyst transfers in poorer prognosis patients, we have relied on our own and outside experts’ clinical judgment to determine that a non-inferiority margin of 10% is clinically acceptable. We used data from Boston IVF to estimate the live birth rate per transfer for cleavage-stage embryos. Among women with ≤5 zygotes on day 1, approximately 24% achieved a live birth after a day 3 transfer. Given that many of these cycles will not result in cryopreservation of embryos and subsequent frozen embryo transfer, it is reasonable to assume a live birth rate per cycle of approximately 25% for p.f.d. 3 transfers. Assuming a 25% live birth rate in both groups, a non-inferiority margin of 10%, a one-sided significance level of 0.025, and 80% power, a sample size of 296 per group is required. To allow for up to 10% of patients to withdraw from the study after randomization or be lost to follow-up, we aim to enroll 329 participants per group, yielding a total sample size of 658 participants. We estimate that there were 710 eligible patients in 2018 across all sites. Assuming 50% of eligible patients will consent to study participation, we anticipate an enrollment period of 22 months. Given that patients store their frozen embryos at our facilities we expect minimal loss to follow-up.

### Data collection and statistical analysis

For all women who consent to participate, the following data will be collected (i) the number and quality of transferred cleavage and blastocyst stage embryos according to the Gardner grading criteria [17] (ii) pregnancy test result 2 weeks after egg retrieval or 9 days after frozen embryo transfer, (iii) if pregnancy test is positive, we will perform an obstetric transvaginal ultrasound at 7–8 weeks gestational age to confirm ongoing pregnancy and multiplicity, (iv) pregnancy outcome (miscarriage, fetal demise, live birth). These data will be entered electronically into patients’ medical records as part of routine clinical care and collected by the study team for analysis. A password system will be utilized to control access to the study data. A complete backup of the primary database will be performed twice a month. All data reports will be prepared such that no individual participant can be identified.

Both intention-to-treat and per-protocol analyses are noted in the RCT reporting guidelines (CONSORT) as valid approaches. For this study, we will perform an intention-to-treat analysis because it is the gold standard for RCTs, even with crossover, withdraw and loss to follow-up. We also will perform a per-protocol analysis as it is a valid statistical approach and will serve as a sensitivity analysis. Descriptive data will be presented as proportion, mean with standard deviation or median with interquartile range. Comparisons will be made using Chi-square or Fisher’s exact tests for categorical variables and parametric or non-parametric tests for continuous variables based on data distribution. We will use log-binomial regression to estimate risk ratios (RR) and 95% confidence intervals for the primary and secondary outcomes. While we anticipate that randomization will balance the distribution of measured and unmeasured potential confounders in the two study arms, if this is not the case, we will assess the influence of potential confounders as needed. In addition, we will perform pre-specified subgroup analysis among participants with ≤2 versus 3–5 zygotes on p.f.d 1, as well as participants with poor versus good quality embryos undergoing single embryo transfer. We will base analyses of the heterogeneity of treatment effects for these subgroups on a statistical test for interaction [18, 19]. This interaction test examines the extent to which any observed difference across subgroups may be attributed to chance variation. All data will be analyzed with SAS 9.4 (SAS Institute Inc., Cary, NC, USA).

**Fig. 3:**
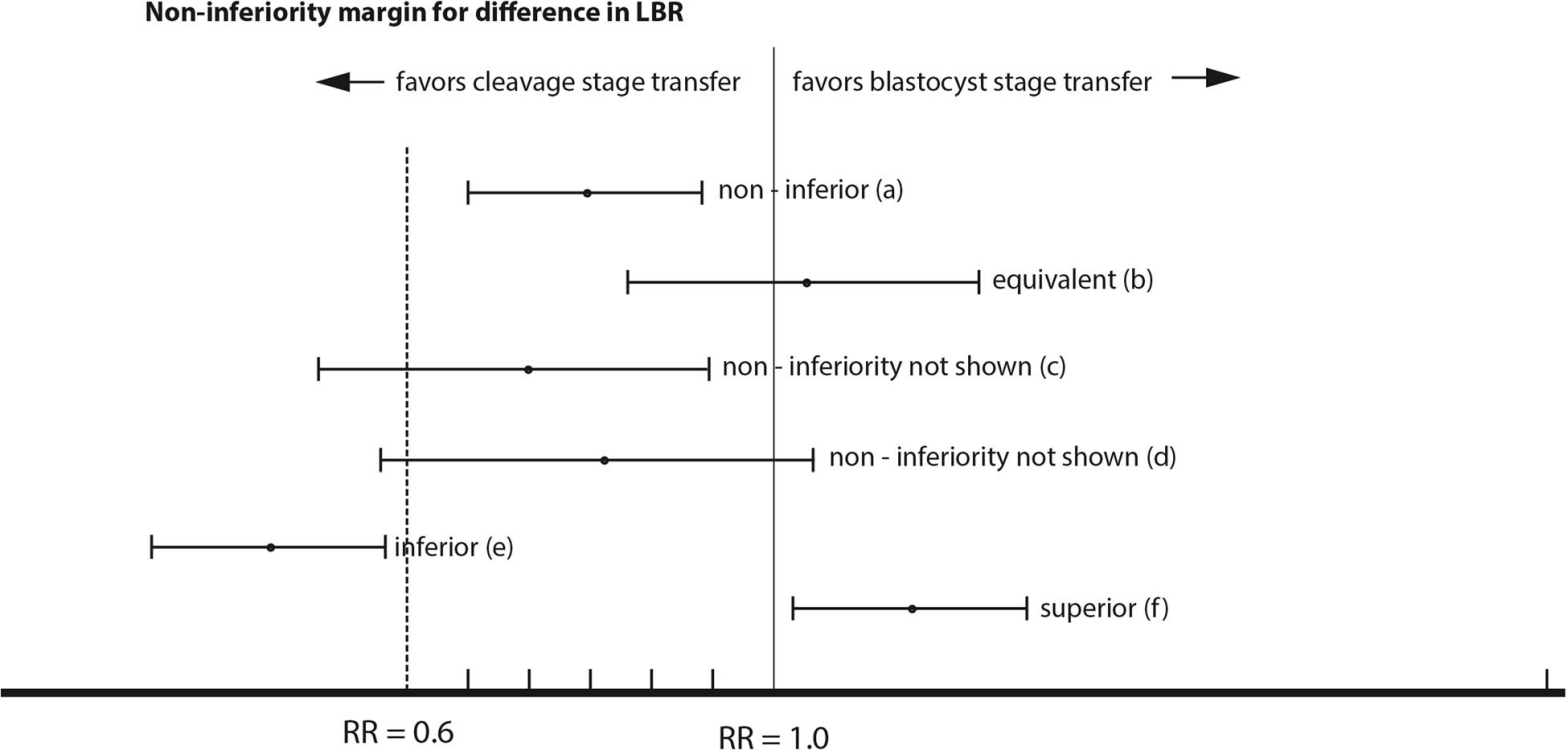
Possible outcomes of the PRECiSE non-inferiority RCT. The non-inferiority margin is set to a 10% absolute difference in live birth rate, which corresponds to a risk ratio (RR) of 0.6 for p.f.d 5 versus p.f.d.3 embryo transfer. **a** the lower bound of the confidence interval (CI) lies above the non-inferiority margin of 0.6: p.f.d. 5 is non-inferior to p.f.d. 3 embryo transfer (**b**) the lower bound of the CI lies above the non-inferiority margin and the CI includes the null value (RR = 1.0): p.f.d. 5 is non-inferior and equivalent to p.f.d. 3 embryo transfer (**c**) and (**d**) the CI includes the non-inferiority margin of 0.6: inconclusive (**e**) the upper bound of the CI lies below the non-inferiority margin of 0.6: p.f.d. 5 is inferior to p.f.d. 3 embryo transfer (**f**) the lower bound of the CI lies above the null value (RR = 1.0): p.f.d. 5 is superior to p.f.d. 3 embryo transfer

The primary outcome of the trial is the live birth rate (≥ 22 weeks of gestation) per retrieval among poorer prognosis participants who have a cleavage stage embryo transfer compared with a blastocyst transfer in an intent-to-treat analysis. The intention-to-treat population will include all randomized participants, including those who cross over to the other treatment group (i.e. participants who are randomized to the blastocyst transfer group but in fact receive a cleavage-stage embryo transfer or participants in the cleavage-stage group who request a blastocyst transfer). Assuming a 25% live birth rate for cleavage stage transfer, a 10% absolute difference in live birth rate between blastocyst and cleavage-stage embryo transfer corresponds to a RR of 0.6 (RR = 0.15/0.25) (Fig. 3). The primary analyses will therefore be interpreted as follows: If the lower bound of the 95% confidence interval (CI) for the difference in live birth rates per retrieval lies to the right of the 10% non-inferiority margin (RR = 0.6), we will have proven non-inferiority of blastocyst transfers at the level of significance α = 0.025; superiority will be demonstrated if the lower bound of the two-sided 95% CI lies fully to the right of 1.0. If the whole 95% CI lies below the non-inferiority margin (RR = 0.6) for the outcome difference the treatment group will be declared “inferior.” If the 95% CI includes the RR = 0.6 noninferiority margin the study results will be deemed inconclusive (Fig. 3) [20].

### Interim data analysis

A Data Safety and Monitoring Board (DSMB) with no direct involvement in the trial will be appointed. The DSMB is independent of the study organizers at Boston IVF and Clinica Eugin and is chaired by Dr. Michele Hacker (Beth Israel Deaconess Medical Center). During the period of recruitment to the study, interim analyses will be supplied, in strict confidence, to the DSMB, together with any other analyses that the committee may request. The role of the DSMB will be to deal with any ethical issues that may arise while the trial is in progress and to review the interim analysis. An interim analysis of clinical pregnancy rates will be conducted and reviewed by the DSMB after 50% of the study participants have been randomized.

### Stopping the trial

The DSMB will be asked to give advice regarding stopping the trial if they have evidence of a meaningful advantage or disadvantage for one of the treatment groups and they consider that the results are likely to affect clinical practice. The following guidelines are proposed for the DSMB to recommend stopping the trial or temporarily suspending recruitment if any of the following are observed at the time of the interim analysis: (1) the difference in clinical pregnancy rate exceeds 20% in one fresh transfer group compared with the other; a difference between the two groups will be considered significant if the *p* value for the difference is less than 0.001 (2) there are notably more adverse events, such as miscarriage or multiple pregnancy, in one ET group compared with the other (3) recruitment is not proceeding at rates that will allow the study to reach its target sample size in a reasonable timeframe. It should be noted that if there appears to be an unexpectedly high number of cancelled fresh embryo transfers in the day 5 transfer group, any one of the study institutional review boards may also temporarily or permanently halt the study at any time. If the study is stopped temporarily or permanently for any reason, follow-up of participants already enrolled will continue as originally scheduled and all participants already enrolled will receive continued care, appropriate to their clinical condition and circumstances, in line with each site’s standard practice.

### Duration of project

It is anticipated that the trial recruitment and all follow-up can be completed in approximately 31 months. Recruitment will begin in January 2020 after trial procedures have been tested, study staff trained and materials distributed to all study sites within the Boston IVF and Clinica Eugin networks.

## Discussion

The PRECiSE trial aims to determine whether transfer of cleavage stage embryos in patients with ≤5 available embryos results in similar pregnancy outcomes compared to the culture of all embryos to p.f.d. 5 in vitro followed by blastocyst transfer. In addition, we will evaluate potential negative consequences of cleavage stage embryo transfer (miscarriage and multiple pregnancy) and blastocyst transfer (cycle cancellation). The need for this RCT is clear, because previous trials have not assessed pregnancy outcomes in poorer prognosis patients and cleavage stage embryo transfer is routinely performed in this subgroup at many IVF centers. The PRECiSE trial differs from previous trials in that it will report rates of live birth *per retrieval* together with the incidence of adverse events such as miscarriage and multiple pregnancies.

This pragmatic trial replaces a clinical decision based on empiric physician preference and experience and as a result does not interfere with the logistics of routine IVF care. However, the main challenge for this trial is the relatively low number of patients with ≤5 zygotes on day 1 after fertilization. This is overcome by the large numbers of IVF cycles per year at the participating IVF centers and the inclusion of national and international satellite offices within our network. While patient populations across study sites may vary, stimulation protocols, embryo culture protocols and pregnancy rates are comparable, making the findings generalizable. Another challenge of this study is the possible higher dropout rate for patients with only 1–2 embryos on p.f.d. 1. This is mainly driven by the concern for ‘having nothing to transfer’. This will be partially mitigated by detailed counseling of patients in this group about the absence of evidence for benefit either way.

If the interim analysis indicates a difference in pregnancy rates per retrieval above the 10% inferiority margin we will change the design of the study to a superiority RCT. If no clinically significant differences in pregnancy rates and secondary outcomes are identified, this trial will have important implications for the clinical practice of IVF and may facilitate the adoption of routine blastocyst culture for all patients. This could result in simplified laboratory protocols and reduced costs, as well as higher rates of single embryo transfers and potentially a reduced incidence of multiples and miscarriage.

## Data Availability

Final study datasets will be stored locally and securely at Beth Israel Deaconess Medical Center for long-term storage and access. Anonymized participant-level data will be made available by request on a case-by-case basis.

## Abbreviations

ART: Assisted reproductive technology
ASRM: American Society of Reproductive Medicine
CI: Confidence interval
DSMB: Data Safety and Monitoring Board
FET: Frozen embryo transfer
ITT: Intent-to-treat
IVF: In vitro fertilization
p.f.d.: Post fertilization day
RCT: Randomized controlled trial
